# Prolonged light exposure induces widespread phase shifting in the circadian clock and visual pigment gene expression of the *Arvicanthis ansorgei* retina

**Published:** 2013-05-21

**Authors:** Corina Bobu, Cristina Sandu, Virginie Laurent, Marie-Paule Felder-Schmittbuhl, David Hicks

**Affiliations:** Department of Neurobiology of Rhythms, Institute for Cellular and Integrative Neurosciences, Strasbourg, France

## Abstract

**Purpose:**

Prolonged periods of constant lighting are known to perturb circadian clock function at the molecular, physiological, and behavioral levels. However, the effects of ambient lighting regimes on clock gene expression and clock outputs in retinal photoreceptors—rods, cones and intrinsically photosensitive retinal ganglion cells—are only poorly understood.

**Methods:**

Cone-rich diurnal rodents (Muridae: *Arvicanthis ansorgei*) were maintained under and entrained to a 12 h:12 h light-dark cycle (LD; light: ~300 lux). Three groups were then examined: control (continued maintenance on LD); animals exposed to a 36 h dark period before sampling over an additional 24 h period of darkness (DD); and animals exposed to a 36 h light period before sampling over an additional 24 h period of light (~300 lux, LL). Animals were killed every 3 or 4 h over 24 h, their retinas dissected, and RNA extracted. Oligonucleotide primers were designed for the *Arvicanthis* clock genes *Per1*, *Per2*, *Cry1*, *Cry2*, and *Bmal1*, and for transcripts specific for rods (rhodopsin), cones (short- and mid-wavelength sensitive cone opsin, cone arrestin, arylalkylamine N-acetyltransferase) and intrinsically photosensitive retinal ganglion cells (melanopsin). Gene expression was analyzed by real-time PCR.

**Results:**

In LD, expression of all genes except cone arrestin was rhythmic and coordinated, with acrophases of most genes at or shortly following the time of lights on (defined as zeitgeber time 0). Arylalkylamine N-acetyltransferase showed maximal expression at zeitgeber time 20. In DD conditions the respective profiles showed similar phase profiles, but were mostly attenuated in amplitude, or in the case of melanopsin, did not retain rhythmic expression. In LL, however, the expression profiles of all clock genes and most putative output genes were greatly altered, with either abolition of daily variation (mid-wavelength cone opsin) or peak expression shifted by 4–10 h.

**Conclusions:**

These data are the first to provide detailed measures of retinal clock gene and putative clock output gene expression in a diurnal mammal, and show the highly disruptive effects of inappropriate (nocturnal) lighting on circadian and photoreceptor gene regulation.

## Introduction

Living organisms possess an intrinsic circadian timekeeping system to synchronize their physiology with the environment. The retina has a double interest in this respect, since on the one hand, light is the most powerful “zeitgeber,” acting though intrinsically photosensitive retinal ganglion cells (ipRGCs) projecting to the central circadian clock in the suprachiasmatic nucleus (SCN) [1], and on the other, the retina exhibits numerous rhythmic physiological processes of its own, including melatonin synthesis [2], ion channel sensitivity [3,4], visual pigment synthesis [5], and phagocytosis of shed photoreceptor (PR) outer segments (OSs) [6]. PRs are highly metabolically active cells, undergoing constant membrane renewal such that the OSs are replaced entirely within 7–10 days [7]. This turnover is composed of several sequential, synchronized steps: RNA synthesis of visual pigments, protein translation and transport, new membrane formation at the apical surface of the OSs, and removal of aged damaged membrane from the distal end. This latter process is achieved through phagocytosis of shed membrane by the apposing retinal pigmented epithelium [8]. Each step of this renewal process is tightly regulated, and errors in any one of them may lead to PR breakdown and death. For example, rhodopsin transcription levels are controlled precisely, with under- [9] and overexpression [10] leading to PR degeneration. Mutations in the mer receptor tyrosine kinase (MERTK) receptor essential for PR phagocytosis lead to retinal breakdown in animals [11] and humans [12]. A great deal of effort has been made to define the environmental and molecular control mechanisms of these different processes. Visual pigment synthesis and phagocytosis are both known to be controlled by light and/or circadian clocks [5,6,13-15]. There is evidence that some of these activities are regulated by (an) endogenous retinal clock(s), since cultured retinas continue to synthesize melatonin in a rhythmic manner [16] and optic nerve section does not perturb phagocytosis [17]. However, the precise cellular localization of retinal clocks, and more importantly, their functional organization at the tissue level, are still unknown. In mammals, these phenomena have generally been studied in rats and mice, which are both naturally nocturnal species. Consequently, there is a lack of information on clock activity in the retinas of diurnal species, and especially with respect to cone PRs, which are poorly represented in mice and rat retinas [18,19].

We used a diurnal rodent, *Arvicanthis ansorgei* (Muridae), which we showed previously contains tenfold more cone PRs than mice [14], to investigate cellular and molecular rod, cone, and ipRGC responses to varying lighting regimes. We observed previously that rhythmic phagocytosis in PRs continues unabated when animals are placed in constant darkness (DD), but that maintenance in light (LL) leads to extensive perturbation of phagocytosis and loss of rhythmicity [20,21]. The present study was designed to determine whether changes in the light environment also altered other aspects of PR turnover, notably visual protein messenger RNA (mRNA) synthesis, and whether any modifications could be correlated with shifts in retinal clock gene expression. The data showed that as for phagocytosis, LL conditions greatly perturb the rhythmic expression of multiple PR and clock genes.

## Methods

### Animal care and handling

All animal experimentation was performed according to institutional and national guidelines, and adhered to the Association for Research in Vision and Ophthalmology Guidelines for Use of Animals, and to the European Communities Council Directive of 24 November 1986 (86/609/EEC) and the Animal Use and Care Committee from Strasbourg. The experimental procedures were covered by an authorization to perform small animal experimentation (Veterinary Section, Ministry of Agriculture, visa 67–132). This study was conducted using Sudanian unstriped grass rats (*Arvicanthis ansorgei*), born and reared in our Chronobiotron animal facilities (UMS 3415) from individuals captured in southern Mali in 1998 [22]. Adult (4–16 months of age) *Arvicanthis ansorgei* were housed in individual cages under standard 12h:12h light-dark cycles (LD; light at 300 lux), lights on at 7 AM (defined as zeitgeber time [ZT] 0), lights off at 7 PM, with free access to food (standard rat chow) and water. For the different analyses, we made sure that each sample contained a mix of young and older retinas.

**Figure 1:**
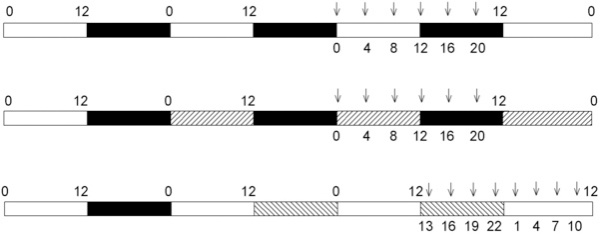
Schematic diagram showing time schedule of experiments and sampling points. The bars show the paradigms used in light and dark (LD) conditions (alternating white [light, 300 lux, 12 h] and black [dark, 12 h] bars]; DD (alternating right hatched [subjective day] and black [subjective night] bars); and LL (alternating white [subjective day] and left hatched [subjective night] bars]. Arrows indicate time points at which animals were killed and examined.

For LD studies (Figure 1, first line), animals (n=3–6 per time point) were taken every 4 h through a complete 24 h period, starting at ZT1. They were anesthetized by isoflurane inhalation and decapitated; the cornea of each eye was slit with a clean scalpel blade, the lens and vitreous were discarded, and the retina was collected and snap frozen individually in sterile Eppendorf tubes in liquid nitrogen. For constant dark studies (DD) (Figure 1, second line), animals previously housed under the standard LD condition were placed in total darkness for 36 h before collection of samples as above (i.e., animals left for one complete cycle of subjective day and night, retinas collected starting on the second subjective day under dim red light every 4 h through a complete 24 h period, n=6 per time point; first collection performed at circadian time [CT] 0). For prolonged light (LL) studies (Figure 1, third line), animals were left in permanent 300 lux white light for 36 h before collection of samples (i.e., animals left for one complete cycle of subjective night and day, retinas collected starting on the second subjective night every 3 h through a complete 24 h period, n=4 per time point; first collection performed at CT13). For figures showing gene expression profiles under LL, time points are displayed according to time of day, starting at CT1.

### Real-time quantitative polymerase chain reaction

Total RNA was extracted using the Absolutely RNA Miniprep kit (Stratagene, La Jolla, CA). Briefly, isolated *Arvicanthis* retinas (snap frozen in liquid nitrogen and stored at −80 °C) were homogenized using a 1 ml syringe and a 27 gauge needle. Total RNA was eluted with 30 µl elution buffer (10 mM Tris-HCl pH 7.5, 0.1 mM EDTA). RNA concentration and purity (A_260_/A_280_ and A_260_/A_230_) were measured using a NanoDrop ND-1000 V 3.5 Spectrophotometer (NanoDrop Technologies, Wilmington, DE). Integrity of the RNA was assessed by visualization of the 28S and 18S ribosomal RNA bands by agarose gel electrophoresis or by using the 2100 Bioanalyzer (Agilent Technologies, Santa Clara, CA; RNA integrity numbers were between 6 and 9).

Total RNA (500 ng) was reverse transcribed into first strand cDNA in the presence of 200 ng of random hexamer primers (Fermentas) using 200 U of RevertAid H Minus M-MuLV Reverse Transcriptase (Fermentas, Burlington, Canada) at 42 °C for 60 min. All cDNA samples were stored at −80 °C. Quantitative PCR primer sequences (Table 1) were designed using the Primer Express V 3.0 software (Applied Biosystems, Foster City, CA), based on previously published sequences from *Arvicanthis ansorgei* brain and muscle aryl hydrocarbon receptor nuclear translocator like (*Bmal*) 1, period (*Per*) 2, cryptochrome (*Cry*) 1, and *Cry2* genes, from *Arvicanthis niloticus* *Per1* and arylalkylamine N-acetyltransferase (*Aanat*) genes and on partial cloning and sequencing of rhodopsin (*Opn2*), mid- and short wavelength cone opsins (*Opn1mws* and *Opn1sws* respectively), cone arrestin (*Arr3*), and melanopsin (*Opn4*) genes from *Arvicanthis ansorgei*. For the latter, reverse transcription (RT)–PCR was performed on RNA extracted from *Arvicanthis ansorgei* retina with degenerate primers based on known mammalian sequences from these genes and sequences of cDNA fragments released into GenBank. Position of primers for quantitative PCR was determined so as to overlap with putative exon/intron boundaries, as predicted from mice genomic sequences, and their specificity was confirmed by basic local alignment search tool (BLAST) searching. The length of the amplicons was kept under 200 bp (55–160 bp), and the melting/annealing temperature (T_m_) of all primers was optimized to 60 °C. The designed primers (high performance liquid chromatography [HPLC] purified) were synthesized by Invitrogen (Carlsbad, CA). Details of the primers and the GenBank Accession Numbers are given in Table 1.

**Table 1:** Primer sequences.

Gene	GenBank	Arvicanthis species	Forward	Reverse	bp
*b-Actin*	EU862078	*Arvicanthis ansorgei*	CTGCTGCATCCTCTTCCTCTCT	CCACAGGATTCCATACCCAAA	133
*Opn2*	EU862075	*Arvicanthis ansorgei*	TCGTTGGCTGGTCCAGGTA	TGTAGTAGTCAATCCCACATGAACAC	63
*Opn1mws*	EU862074	*Arvicanthis ansorgei*	TGGCAATGTGAGATTTGATGCT	CCAGACCCAGGAGAAGACGAT	61
*Opn1sws*	EU862076	*Arvicanthis ansorgei*	AGCGCAGCAGCAAGAGTCA	ATGGCTCACCTCCCGTTCAG	55
*Arr3*	EU862077	*Arvicanthis ansorgei*	CATGCGCAGCTTCTTTCTGTC	ATAGCTTCTCCATGGTAATGAAC	84
*Opn4*	KC150901	*Arvicanthis ansorgei*	CAGGGATGCTGGGCAATCT	GTGTCCGCAGGCCTCTGTT	63
*Bmal1*	AY225378	*Arvicanthis ansorgei*	GACACTGAGAGGTGCCACCAA	CCATCTGCTGCCCTGAGAAT	102
*Per1*	AY817662	*Arvicanthis niloticus*	CCACTGAGAGCAGCAAGAGTACA	CTGCTGCAGCCACTGGTAGA	121
*Per2*	AY225379	*Arvicanthis ansorgei*	TCACCGTAGGAGATCCGGAAT	TTTCTGCAACAGGTGCTTCCT	103
*Cry1*	AY196136	*Arvicanthis ansorgei*	TGAAGGTCTTTGAGGAATTACTGCT	CGCCTAATATAGTCTCCATTGGGA	160
*Cry2*	AY196137	*Arvicanthis ansorgei*	TGACGAGCTGCTCCTGGAT	GCAGGTATCGCCGGATGTA	157
*Aanat*	AF317891	*Arvicanthis niloticus*	AGAGCTGTCACTGGGCTGGTT	CGACTCCTGAGTAAGTCTCTCCTTGT	91

GenBank accession numbers and anticipated size (bp) of the amplicons for the studied genes. *Arvicanthis* species from which the primers were designed is specified.

Real-time quantitative PCR was performed using the 7300 Real Time PCR System (Applied Biosystems) and fluorescent SYBR Green I chemistry. The PCR conditions were: 1 x Power SYBR Green (Applied Biosystems), 900 nM forward primer, 900 nM reverse primer (Invitrogen), and 1 µl of cDNA in a total volume of 20 µl. The PCR program was as follows: denaturation at 95 °C for 10 min, followed by 40 cycles of denaturation at 95 °C for 15 s and annealing-elongation at 60 °C for 1 min. The acquisition of fluorescence data was performed at the end of the elongation step using the 7300 System Sequence Detection Software V 1.3.1 (Applied Biosystems). A dissociation curve was constructed at the end of the PCR run by ramping the temperature of the sample from 60 °C to 95 °C while continuously collecting fluorescence data. The melting profiles indicated a single PCR product and no accumulation of primer dimers. No-template reactions were performed as negative controls for each primer pair. The PCR mix contained the internal passive reference dye 6-carboxyl-X-rhodamine (ROX) for normalization of the eventual non-PCR-related fluorescence fluctuations. Each PCR reaction was done in duplicate, and for each experiment, a dilution curve of pooled cDNA samples was used to calculate the amplification efficiency for each primer set and determine the optimal cDNA dilution according to the manufacturer’s instructions. Real-time PCR data was normalized to *β-actin* and analyzed using the relative quantification model with efficiency corrections according to the Pfaffl method [23,24]. Transcript levels were calculated relative to the sample showing the lowest expression, and which was rescaled to one. All experimental runs were performed as sample maximization setups on 96 well plates. An interrun calibrator was included on each 96 well plate.

### Statistics

Results are presented as means ± standard error of the mean and the first time point (ZT0 in LD, CT0 in DD, CT1 in LL) is double plotted at the end of the 24 h cycle. Statistically significant differences among different ZT or CT groups were analyzed using the one-way analysis of variance (ANOVA) and post hoc tests (Bonferroni or Tukey test; Statistica 8.0, StatSoft Inc., Tulsa, OK) on the normalized data. Gene expression rhythmicity was analyzed using the cosinor method (Sigmaplot V 10.0, Systat Software Inc., San Jose, CA), by fitting the 24 h data to a cosine curve [25].

## Results

### Transcription of visual pigment genes is strongly affected by lighting conditions

**Figure 2:**
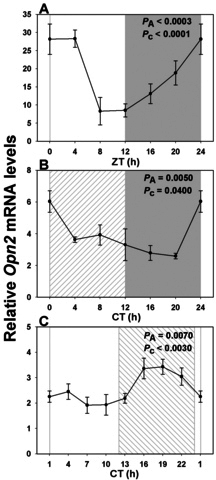
Expression profile of rod-specific rhodopsin transcript over a single 24 h period under distinct lighting conditions. **A**: In a 12 h light: 12 h dark cycle (LD) a rhythmic pattern was observed with maximal expression close to “dawn” (night/day transition), and a nadir 12 h later (n=3–6 per time point). **B**: Rhodopsin expression profile was similar in constant dark (DD; n=6 per time point). **C**: In constant light (LL) there was a large phase shift, such that peak values now occurred during the subjective night (CT19; n=4 per time point). Illumination conditions are depicted as solid white (day) and solid gray (night) areas in LD, right hatched (subjective day) and solid gray (subjective night) areas in DD, and solid white (subjective day) and left hatched (subjective night) areas in constant light LL. Animals were killed every 3 or 4 h over a 24 h period, and RNA extracted from retinal tissue. RNA expression levels were quantified by real-time PCR. One-way analysis of variance (ANOVA) and cosinor levels of significance (P_A_ and P_c_ respectively) are given in the upper right corner of each panel.

Real-time PCR quantification of *Opn2* expression levels every 4 h throughout the LD cycle revealed a highly statistically significant rhythmic profile, with an acrophase centered on the night/day transition point, i.e., ZT0 (Figure 2A and Table 2). The peak-to-trough difference was fourfold. Similar analyses performed during DD showed a largely similar profile with a maximum at CT2.5 and a twofold peak-to-trough difference (Figure 2B and Table 2). In contrast, expression analysis under LL conditions demonstrated that although *Opn2* transcriptional activity was still rhythmic, with 1.8-fold peak-to-trough difference, the acrophase now occurred at CT19 (Figure 2C and Table 2).

**Table 2:** Cosinor and one-way ANOVA statistical analysis of the mRNA levels of the visual pigment and *Aanat* genes in *Arvicanthis* retina in LD, DD and LL conditions.

**Gene**	**COSINOR**			**ANOVA**	
	acrophase (h)	*F-*value	P value	*F-*value	P value
**LD (n=32)**					
*Opn2*	0.19±0.73	*F*_2,29_ 14.00	<0.0001	*F*_5,26_ 6.98	0.0003
*Opn1mws*	1.84±0.80	*F*_2,29_ 10.03	0.0005	*F*_5,26_ 6.08	0.0007
*Opn1sws*	0.84±0.58	*F*_2,29_ 20.80	<0.0001	*F*_5,26_ 12.45	<0.0001
*Arr3*	3.35±2.52	*F*_2,29_ 0.95	0.3995	*F*_5,26_ 2.48	0.058
*Opn4*	1.83±0.70	*F*_2,29_ 12.72	0.0001	*F*_5,26_ 8.35	<0.0001
*Aanat*	19.58±0.39	*F*_2,29_ 53.00	<0.0001	*F*_5,26_ 31.85	<0.0001
**DD (n=36)**					
*Opn2*	2.47±1.44	*F*_2,33_ 3.51	0.0414	*F*_5,30_ 4.30	0.005
*Opn1mws*	0.39±1.32	*F*_2,33_ 4.20	0.0239	*F*_5,30_ 3.31	0.017
*Opn1sws*	1.49±1.06	*F*_2,33_ 6.52	0.0041	*F*_5,30_ 7.54	0.0001
*Arr3*	1.11±1.49	*F*_2,33_ 3.30	0.0495	*F*_5,30_ 3.68	0.01
*Opn4*	3.60±2.17	*F*_2,33_ 1.55	0.2282	*F*_5,30_ 1.62	0.1842
*Aanat*	16.34±1.19	*F*_2,31_ 5.10	0.0122	*F*_5,28_ 6.43	0.0004
**LL (n=32)**					
*Opn2*	19.27±0.82	*F*_2,29_ 10.79	0.0003	*F*_7,24_ 3.80	0.0065
*Opn1mws*	16.53±1.42	*F*_2,29_ 3.58	0.0407	*F*_7,24_ 1.09	0.4034
*Opn1sws*	16.51±0.85	*F*_2,29_ 10.02	0.0005	*F*_7,24_ 3.50	0.01
*Arr3*	16.37±0.72	*F*_2,29_ 14.04	<0.0001	*F*_7,24_ 5.00	0.0013
*Opn4*	15.18±0.82	*F*_2,29_ 10.80	0.0003	*F*_7,24_ 4.75	0.0018
*Aanat*	22.44±1.01	*F*_2,29_ 7.07	0.0032	*F*_7,24_ 2.60	0.0377

**Figure 3:**
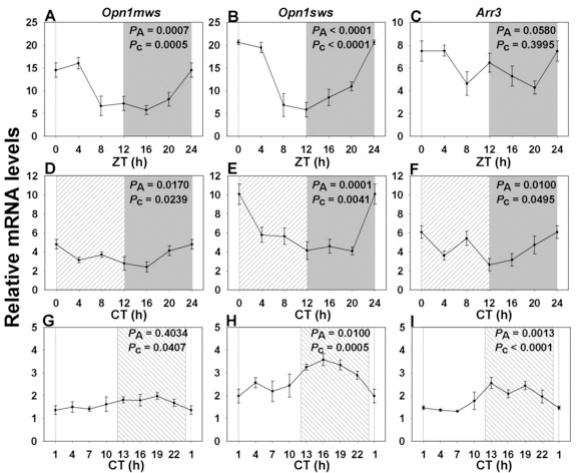
Expression profile of cone vision-related genes over a single 24 h period under distinct lighting conditions. **A**, **D**, **G**: RNA expression levels of *Opn1mws* in a 12 h light: 12 h dark cycle (LD), in constant dark (DD) and in constant light (LL). **B**, **E**, **H**: RNA expression levels of *Opn1sws* in LD, DD and LL. **C**, **F**, **I**: RNA expression levels of *Arr3* in LD, DD and LL. In LD (n=3–6 per time point) both *Opn1mws* and *Opn1sws* transcripts showed rhythmic patterns with maximal expression at or closely following the night/day transition, and a nadir 12 h later (**A**, **B**). *Arr3* expression did not fit a cosinor function (**C**). The shapes of the curves were mostly similar under DD (**D**: *Opn1mws*, **E**: *Opn1sws*, **F**: *Arr3*; n=6 per time point). However, LL conditions led to large phase shifts, with maxima in the early to middle night (**G**: *Opn1mws*, **H**: *Opn1sws*, **I**: *Arr3*; n=4 per time point). Illumination conditions are depicted as solid white (day) and solid grey (night) areas in LD, right hatched (subjective day) and solid grey (subjective night) areas in constant dark (DD) and solid white (subjective day) and left hatched (subjective night) areas in constant light (LL). Animals were killed every 3 or 4 h over a 24 h period, and RNA extracted from retinal tissue. RNA expression levels were quantified by real-time PCR. One-way analysis of variance (ANOVA) and cosinor levels of significance (P_A_ and P_c_ respectively) are given in the upper right corner of each panel.

As seen for *Opn2*, the expression profiles for *Opn1mws* and *Opn1sws* were rhythmic in LD (Table 2), with their maxima occurring shortly after the night-day transition, i.e., ZT2 for *Opn1mws* (Figure 3A) and ZT1 for *Opn1sws* (Figure 3B), and peak-to-trough ratios of 3 and 4, respectively. *Arr3* transcriptional activity was not rhythmic by cosinor analysis, although there was still a trend to daily variation by ANOVA (Figure 3C and Table 2). Rhythmic *Opn1mws* and *Opn1sws* expression was maintained with similar profiles of maxima and minima in DD (Table 2), with a maximum range of twofold between peak and trough (*Opn1mws*, peak CT0: Figure 3D; *Opn1sws*, peak CT1.5: Figure 3E). In DD, *Arr3* expression reached statistical significance both by ANOVA and cosinor analysis (Table 1), with an acrophase at CT1 (Figure 3F). In LL conditions, as for *Opn2*, rhythmic expression of *Opn1sws* was maintained (Table 2), but with a very large (8 h) advance in the peak expression value (peak CT16.5: Figure 3H) with respect to LD and reduced amplitude. *Opn1mws* behaved rather similarly, with a 9 h advance of the peak phase (peak CT16.5: Figure 3G and Table 2), but did not show significant variation by ANOVA, even if it proved rhythmic following cosinor analysis. Furthermore, *Arr3* was rhythmic under LL conditions, with an expression peak at CT16 (Figure 3I and Table 2) and a peak-to-trough difference of 1.7-fold, which is similar to that for opsin transcripts.

### Daily profile and lighting effects on melanopsin expression in *Arvicanthis ansorgei*

**Figure 4:**
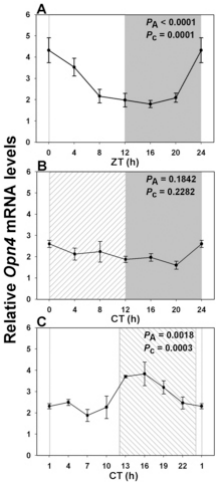
Expression profile of intrinsically photosensitive retinal ganglion cell-specific melanopsin transcript over a single 24 h period under distinct lighting conditions. **A**: In a 12 h light: 12 h dark cycle (LD) there was a rhythmic pattern with maximal expression close to “dawn” (night/day transition), and a nadir 12 h later (n=3–6 per time point). **B**: Melanopsin expression profile was attenuated in constant dark (DD) and did not attain significance (n=6 per time point). **C**: However, in constant light (LL) there was again a large phase shift, such that peak values now occurred in early night (CT15; n=4 per time point). Illumination conditions are depicted as solid white (day) and solid grey (night) areas in LD, right hatched (subjective day) and solid grey (subjective night) areas in constant dark (DD) and solid white (subjective day) and left hatched (subjective night) areas in constant light (LL). Animals were killed every 3 or 4 h across the 24 h period, and RNA extracted from retinal tissue. RNA expression levels were quantified by real time PCR. One-way analysis of variance (ANOVA) and cosinor levels of significance (P_A_ and P_c_ respectively) are given in the upper right corner of each panel.

We employed a standard PCR approach to clone the majority of the *Arvicanthis ansorgei Opn4* coding sequence (GenBank accession number KC150901), based on sequence homologies between *Opn4* genes in humans, rats, and the large isoform encoding cDNA initially characterized in mice. *Arvicanthis ansorgei Opn4* mRNA sequence shows high homology to its ortholog in rodents (between 91 and 94% identity, the highest exhibited in the mouse sequence), and the predicted protein shows the expected features [26] of opsins and melanopsin in particular (data not shown). Melanopsin exhibited similar expression patterns and changes in transcription to the conventional visual pigments listed above (Figures 4A-C and Table 2), but rhythmicity was lost in DD. Peak expression was seen at ZT2 in LD conditions and was phase advanced to CT15 in LL. Differences between maximal and minimal values in LD and DD were approximately twofold.

### Constant lighting conditions only weakly perturb Arylalkylamine N-acetyltransferase expression in *Arvicanthis ansorgei*

**Figure 5:**
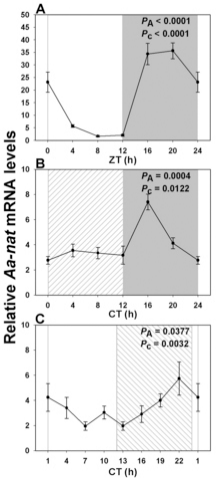
Expression profile of *Aanat* messenger RNA over a single 24 h period under distinct lighting conditions. **A**: In a 12 h light: 12h dark cycle (LD) there was a strongly rhythmic pattern with maximal expression at ZT20, and a nadir 12 h later (n=3-6 per time point). **B**: In constant dark (DD) the profile was attenuated, with a peak at CT16 (n=5-6 per time point). **C**: In constant light (LL) there was only a small phase shift, such that peak values were at CT22 (n=4 per time point). Illumination conditions are depicted as solid white (day) and solid grey (night) areas in LD, right hatched (subjective day) and solid grey (subjective night) areas in constant dark (DD) and solid white (subjective day) and left hatched (subjective night) areas in constant light (LL). Animals were killed every 3 or 4 h across the 24 h period, and RNA extracted from retinal tissue. RNA expression levels were quantified by real time PCR. One-way analysis of variance (ANOVA) and cosinor levels of significance (P_A_ and P_c_ respectively) are given in the upper right corner of each panel.

We also investigated expression profiles of the *Aanat* gene, which encodes the enzyme AANAT, catalyzing the penultimate step of the melatonin synthetic pathway. The profile was very different from those of visual pigments and phototransduction genes: Although expression in LD was again strongly rhythmic (Table 2), it showed a much higher peak-to-trough ratio (~17-fold) than seen in the preceding genes, and the maximal value occurred during the late night at ZT19.5 (Figure 5A). Expression was greatly reduced but still rhythmic in DD (Table 2), with tenfold lower amplitude and a shift in peak expression to CT16 (Figure 5B). While still weakly rhythmic in LL (Table 2), in contrast to the other genes studied, there was a smaller shift in the peak value of *Aanat* expression, to approximately CT23 (Figure 5C).

### Constant light leads to phase reversal of retinal clock gene expression

**Figure 6:**
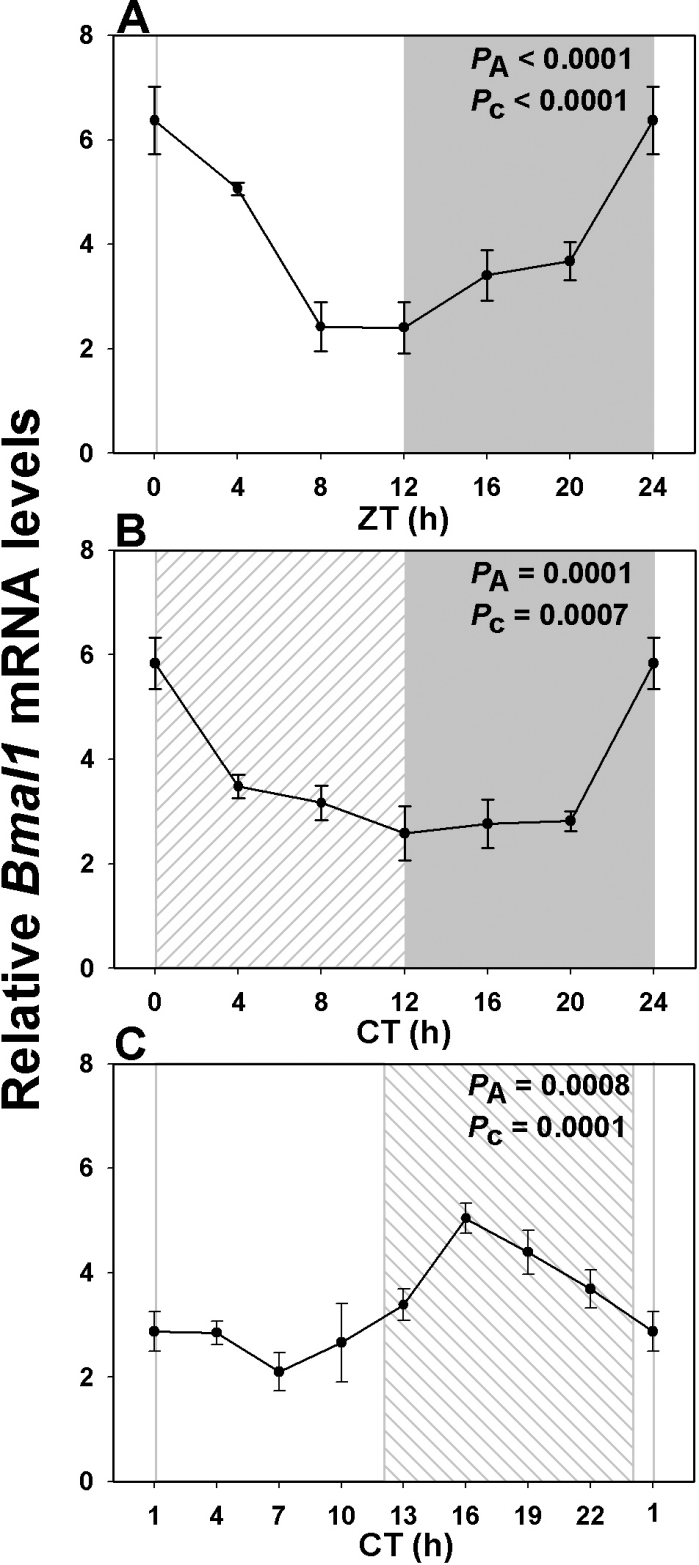
Expression profile of core clock gene *Bmal1* over a single 24 h period under distinct lighting conditions. **A**: In a 12 h light: 12 h dark cycle (LD) *Bmal1* exhibited a rhythmic expression pattern with the peak value shortly after light onset (n=3-6 per time point). **B**: Rhythmicity was maintained although dampened in constant dark (DD) (n=6 per time point). **C**: However constant light (LL), as with the other genes, led to a large phase shift, with maximal values now occurring at CT18 (n=4 per time point). Illumination conditions are depicted as solid white (day) and solid grey (night) areas in LD, right hatched (subjective day) and solid grey (subjective night) areas in constant dark (DD) and solid white (subjective day) and left hatched (subjective night) areas in constant light (LL). Animals were killed every 3 or 4 h across the 24 h period, and RNA extracted from retinal tissue. RNA expression levels were quantified by real time PCR. One-way analysis of variance (ANOVA) and cosinor levels of significance (P_A_ and P_c_ respectively) are given in the upper right corner of each panel.

**Figure 7:**
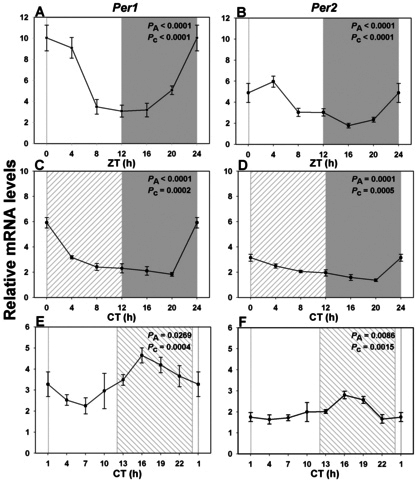
Expression profile of the negative feedback loop *Per* transcripts over a single 24 h period under distinct lighting conditions. **A**, **C**, **E**: RNA expression levels of *Per1* in a 12h light: 12 h dark cycle (LD), in constant dark (DD) and in constant light (LL). **B**, **D**, **F**: RNA expression levels of *Per2* in LD, DD, and LL. Each gene showed a rhythmic pattern with maximal expression at or closely following night/day transition in LD (n=3-6 per time point) and DD (n=6 per time point; **A**, **C**: *Per1*, **B**, **D**: *Per2*). LL conditions led to large phase shifts, with maxima in the early to middle night (**E**: *Per1*, **F**: *Per2*; n=4 per time point). Illumination conditions are depicted as solid white (day) and solid grey (night) areas in LD, right hatched (subjective day) and solid grey (subjective night) areas in constant dark (DD) and solid white (subjective day) and left hatched (subjective night) areas in constant light (LL). Animals were killed every 3 or 4 h across the 24 h period, and RNA extracted from retinal tissue. RNA expression levels were quantified by real time PCR. One-way analysis of variance (ANOVA) and cosinor levels of significance (P_A_ and P_c_ respectively) are given in the upper right corner of each panel.

**Figure 8:**
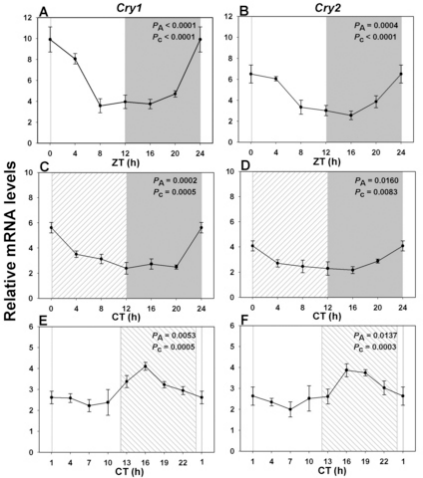
Expression profile of the negative feedback loop *Cry* transcripts over a single 24 h period under distinct lighting conditions. **A**, **C**, **E**: RNA expression levels of *Cry1* in a 12 h light: 12 h dark cycle (LD), in constant dark (DD) and in constant light (LL). **B**, **D**, **F**: RNA expression levels of *Cry2* in LD, DD, and LL. Each gene showed a rhythmic pattern with maximal expression at or closely following night/day transition in LD (n=3-6 per time point) and DD (n=6 per time point; **A**, **C**: *Cry1*, **B**, **D**: *Cry2*). LL conditions led to large phase shifts, with maxima in the early to middle night (**E**: *Cry1*, **F**: *Cry2*; n=4 per time point). Illumination conditions are depicted as solid white (day) and solid grey (night) areas in LD, right hatched (subjective day) and solid grey (subjective night) areas in constant dark (DD) and solid white (subjective day) and left hatched (subjective night) areas in constant light (LL). Animals were killed every 3 or 4 h across the 24 h period, and RNA extracted from retinal tissue. RNA expression levels were quantified by real time PCR. One-way analysis of variance (ANOVA) and cosinor levels of significance (P_A_ and P_c_ respectively) are given in the upper right corner of each panel.

To see whether these light-induced alterations in PR (rod, cone, and ipRGC) gene transcription could be correlated with clock gene expression, we also analyzed the *Arvicanthis* homologs of five core clock genes: *Bmal1*, *Per1*, *Per2*, *Cry1*, and *Cry2*. All five genes exhibited statistically significant rhythmic expression under LD cycles, with acrophases around ZT0, 1, 3, 1, and 1.5 respectively (Figure 6A, Figure 7A-B, Figure 8A-B and Table 3). Amplitudes of peak-to-trough variations were from 1.6- to fourfold. Profiles were attenuated (maximal difference of 2.5-fold) but still rhythmic with similar temporal patterns under DD (*Bmal1*: CT1; *Per1*: CT1; *Per2*: CT3; *Cry1*: CT1; *Cry2*: CT0; Figure 6B, Figure 7C-D, Figure 8C-D and Table 3). As seen for visual transduction–related genes, however, these profiles were all greatly altered under LL, with phase advances of 5.5 to 10.5 h (i.e., maxima around CT18, 18, 16, 17, and 18 for *Bmal1*, *Per1*, *Per2*, *Cry1*, and *Cry2* respectively: Figure 6C, Figure 7E-F, Figure 8E-F and Table 3). The maximal variation between peak and trough values under LL was twofold.

**Table 3:** Cosinor and one-way ANOVA statistical analysis of the mRNA levels of the core clock genes in *Arvicanthis* retina in LD, DD and LL conditions.

**Gene**	**COSINOR**			**ANOVA**	
	acrophase (h)	*F-*value	P value	*F-*value	P value
**LD (n=32)**					
*Bmal1*	0.16±0.70	*F*_2,29_ 15.02	<0.0001	*F*_5,26_ 12.50	<0.0001
*Per1*	1.21±0.51	*F*_2,29_ 25.94	<0.0001	*F*_5,26_ 15.54	<0.0001
*Per2*	3.23±0.65	*F*_2,29_ 14.07	<0.0001	*F*_5,26_ 9.60	<0.0001
*Cry1*	1.16±0.63	*F*_2,29_ 16.94	<0.0001	*F*_5,26_ 14.05	<0.0001
*Cry2*	1.64±0.68	*F*_2,29_ 14.13	<0.0001	*F*_5,26_ 6.72	0.0004
**DD (n=36)**					
*Bmal1*	1.00±0.89	*F*_2,33_ 9.03	0.0007	*F*_5,30_ 9.76	0.0001
*Per1*	1.36±0.82	*F*_2,33_ 10.88	0.0002	*F*_5,30_ 26.18	<0.0001
*Per2*	3.10±0.86	*F*_2,33_ 9.75	0.0005	*F*_5,30_ 10.54	0.0001
*Cry1*	1.35±0.86	*F*_2,33_ 9.65	0.0005	*F*_5,30_ 11.00	0.0002
*Cry2*	0.16±1.14	*F*_2,33_ 5.56	0.0083	*F*_5,30_ 3.34	0.016
**LL (n=32)**					
*Bmal1*	17.91±0.67	*F*_2,29_ 16.32	0.0001	*F*_7,24_ 5.47	0.0008
*Per1*	17.86±0.83	*F*_2,29_ 10.58	0.0004	*F*_7,24_ 2.83	0.0269
*Per2*	16.22±0.94	*F*_2,29_ 8.22	0.0015	*F*_7,24_ 3.60	0.0086
*Cry1*	16.99±0.85	*F*_2,29_ 9.98	0.0005	*F*_7,24_ 3.94	0.0053
*Cry2*	18.14±0.83	*F*_2,29_ 10.63	0.0003	*F*_7,24_ 3.28	0.0137

## Discussion

The data presented here are the first to provide daily expression profiles of the principal visual pigment genes in the retina of a diurnal mammal, and further quantify changes in temporal expression induced by differing light exposure. They are also the first to correlate specific output characteristics with multiple molecular components of the circadian clock. They show that i) under LD conditions, expression profiles of the different genes examined (except *Aanat*) appear synchronized to maximal values at or shortly after dawn; and ii) roughly similar profiles are maintained under DD for many of these genes, indicating that they are driven by circadian clock mechanisms; and iii) they are in the majority of cases greatly perturbed by LL.

Rhythms in visual pigment gene expression during the LD cycle have been described in diurnal species such as chicken and zebrafish, with maxima occurring around the day to night transition, and were shown to persist under DD, indicating they are controlled by a circadian clock [27,28]. In contrast, there are very few data on retinal circadian processes in diurnal mammals, which represent a closer analogy to human visual physiology than nocturnal species such as *Mus musculus* and *Rattus norvegicus*. Daily variations in visual pigment gene expression have been reported in the mouse [5,29] and rat [30], with both nocturnal species showing a broad maximum for *Opn2* mRNA transcription at the day/night transition, and a similar profile for *Opn1sws* in mice [5]. Conversely, a genome-wide scan of mouse retina also revealed weak cyclic behavior for *Opn2* and *Opn1sws*, with a morning maximum [31]. The diurnal species examined here shows an exclusively dawn synchronization of several PR behaviors (rod and cone phagocytosis [14,20], visual pigment and phototransduction gene synthesis [present study]), indicating that the daily control of PR turnover is regulated by a unique mechanism. In support of this hypothesis, rhythmic expression of most visual pigments was maintained in DD with peaks at the subjective dawn, as was the rhythm of OS phagocytosis in both rods and cones [20].

In addition to rod and cone PR, a novel class of intrinsically photosensitive retinal ganglion cells (ipRGCs) has been documented [32,33]. These ipRGCs are responsible for mediating non–image forming visual functions of the retina, including photoentrainment [34-36], pupillary constriction [37], and sleep [38]. The visual pigment expressed in these specialized RGCs is melanopsin (opsin 4), a distant member of the opsin family [39]. In mammals, there is a single melanopsin gene, and the protein is restricted to a small subset of RGCs [32]. It has been reported previously that lighting conditions and inherited retinal degeneration strongly affect melanopsin mRNA and protein levels in the rat [40,41]. In rats, melanopsin expression shows rhythmic daily variations with maximal values around the day/night transition zone, and these fluctuations continue relatively unchanged under DD conditions, revealing that melanopsin expression is under the control of a circadian clock [40]. Our data show a very different situation for *Arvicanthis*, with the highest levels of expression in LD seen shortly after dawn, and disappearance of rhythmic changes under DD. A previous study [42] reported daily fluctuations in melanopsin immunoreactivity in mice, with maximal levels at late night (ZT23) and lowest at ZT4, although this was not confirmed by quantitative analysis of short and long *Opn4* transcripts [43]; similar to our data, they saw no fluctuations under DD conditions. Taken together, these data indicate that the daily regulation of melanopsin is opposite between nocturnal and diurnal species, possibly linked to the function of the melanopsinergic system within the contrasting photic niches.

Within the mammalian retina, quantitative analyses of retinal clock gene expression as a function of daily hour have been performed for *Mus musculus* and *Rattus norvegicus*. There is considerable variation among published reports with respect to the rhythmicity of retinal clock gene expression, with some studies indicating cyclic expression of *Bmal1* [30,44,45], *Per1* [44,46-49], *Cry1* [31,44,48,50], and *Cry2* [30,44,48,50], and others reporting no rhythmic expression of the same transcription factors (*Bmal1* [31,47,51]; *Per1* [30,31,52]; *Cry1* [30,53]; *Cry2* [31,53]). *Per2* was persistently seen as rhythmic in all studies, although some authors [54] were unable to demonstrate significant variations for any clock gene within the retina, once corrected for expression levels (which were high in the retina compared to the heart or liver). Our real-time PCR analysis showed significant daily variations for all five clock genes examined, again with broadly similar profiles exhibiting dawn maxima under LD and DD conditions, with a maximal phase delay of 3 h in the case of *Bmal1* and *Per2*. These expression profiles are quite different from those published for retinas of nocturnal rodents: Previous studies using whole or fractionated rat retinas have shown clock gene acrophases to occur predominantly at the day/night transition [44,53]. Still, they have in common the demonstration of rather clustered peaks for all the clock genes examined. Taken together with the global early morning maxima in *Arvicanthis* retinal output genes, the findings suggest differences in retinas from nocturnal versus diurnal species, possibly related to visual physiology and retinal cellular composition. However, the data are also distinct from clock gene profiles seen in *Arvicanthis* SCN [55], in which *Per2* displayed late day (CT8) and *Cry2* and *Bmal1* displayed early night (CT12–18) optima, features common to SCN from rat/mouse species as well.

It should be borne in mind that these measures reflect averaged values from the entire retina, which contains multiple cell types that are thought to be under different phases depending on the cell type [44,47,53]. The retina seems to stand apart compared to other tissues (e.g., liver, pancreas, SCN) in displaying low amplitude and largely overlapping rhythmic expression of clock genes. Since mechanistic understanding of the circadian clock implies phase opposition between *Bmal1* and *Per*/*Cry* [1], this suggests functioning in the retina is distinctly different. This lack of phase opposition has been reported in previous analyses of the retinal circadian clock [44,53,56]. Since clock gene expression was shown to occur in most retinal cell types, the absence of phase opposition might be due to the following: 1) cell-specific molecular clockwork with distinct phases of core clock genes, or 2) cell-specific amplitudes of core clock gene oscillations with predominance of those showing strongest amplitude. Similar observations were made on human peripheral blood mononuclear cells [57], likely also as a result of heterogeneity inherent to the cell population under scrutiny. A recent report indicates the retina is even more complex, since cones were the only retinal cell type showing sustained and rhythmic expression of most core clock genes [58]. It is apparent that although cones represent a mostly homogeneous cell population, the phase relationships between the six clock genes are distinctly clustered. Taken together with previous data showing the presence of a circadian clock within the inner retina [59] or in PR layers [47], this result strongly suggests that organization of the retinal clock is exceptional in comparison to other tissues.

We also chose to examine the gene coding for the enzyme AANAT, involved in melatonin synthesis [60], as a retinal clock output and positive control. This enzyme is present at high levels in the pineal gland and retina [61]. In the latter, in situ hybridization data suggest that it is localized especially to cones in both rodents ([62]; manuscript in preparation) and chickens [63]. However, AANAT is also expressed by other retinal cells within the inner nuclear and ganglion cell layers [64]. There are some indications that retinal *Aanat* is controlled by molecular mechanisms distinct from the pineal gland, and may serve different purposes in the two tissues [65]. *Aanat* is a clock-controlled gene with an E-box in the promoter sequence, driven by BMAL1/CLOCK transcriptional activation [66]. As is also seen in the pineal gland, *Aanat* levels are highest during the night in rat retina [67], and this was also the case in *Arvicanthis* in LD as well as in DD, although with reduced amplitude in the latter. Reduced rhythmic expression under DD seems at odds with the observed continued cyclic synthesis of melatonin under the same constant conditions (e.g., [16]), but this has been seen in several previous studies [44,53,65,68]. It should be further noted that the *Aanat* profile does not cluster to the same phase as the other PR outputs, suggesting that it is regulated by distinct, clock-derived mechanisms.

The principal finding of this study was that visual protein and clock gene expression retained rhythmicity, but was greatly perturbed under prolonged light. For the vision-related genes, this represented phase advances of about 4 h for a rod-specific gene (*Opn2*), 9 h for cone-specific genes (*Opn1mws* and *Opn1sws*), and 10 h for an ipRGC-specific gene (*Opn4*). The relatively larger shifts in cone genes may indicate that prolonged lighting is more disruptive in this population, possibly in relationship with their photosensitive properties, but confirmation of this requires further experimentation using additional rod- and cone-specific genes. On the other hand, LL did not greatly affect the expression maximum for *Aanat*, which indicates that the profile shifts are specific and not an artifact of sampling or amplification methodology. They also confirm that clock pathways regulating *Aanat* expression are different, as in LD and DD, and possibly that opsins can be submitted to additional, light-driven controls. For clock genes, the advances varied from around 6 h for *Bmal1*, 8 h for *Per1*, *Cry1*, and *Cry2*, and an almost complete phase reversal for *Per2*. It is difficult to make a strict correlation between the two sets of profiles for two reasons. First, as previously mentioned, clock gene data are average values for the entire retina, and may be biased by expression levels and rhythms in non-PR populations. For example, *Per* genes appear to be more strongly expressed in the inner than the outer retina (*Per1* [49], *Per2* [46], or both [44]). Second, the putative output genes (except *Aanat*) do not constitute known clock-controlled genes, since they do not possess the E-box motif within their promoters [69]. Hence, they are presumably driven by intermediate transcription factors which remain to be elucidated. Nevertheless, it is clear that the *Arvicanthis* retina behaves differently from the rat retina regarding exposure to prolonged light. Although studies performed under similar conditions to those reported here (a single 24 h cycle of LL) are relatively few, clock gene expression was shown to be dampened [48], as was that of *Aanat* and *Opn4* [70]. This could indicate a global desynchronization of individual oscillators as reported for the SCN. In contrast, in *Arvicanthis*, the rhythmic expression of most genes examined was still sustained, with amplitudes similar to those found in DD, suggesting that extended lighting affects the constitutive oscillators of the retina in a different way, presumably related to retinal adaptation to its photic niche.

We speculate that the interplay between lighting regimes and clock gene expression may underlie the rhythmic profiles of rod and cone turnover. In LD and DD, striking synchrony exists between the acrophases of clock and phototransduction-related gene expression, and the peak of rod and cone shedding [14,20]. This suggests that the retinal circadian clock controls the daily coordination of synthesis and degradation of OSs. Since these processes likely constitute the highest energy expenditure during the 24 h cycle, it can be hypothesized that their temporal association represents a net energy gain compared to if they were spread across the 24 h period. Interestingly, both rod and cone phagocytosis display a second smaller peak around CT19 in DD [20], which cannot be directly correlated with clock gene expression patterns. This secondary phagocytosis burst may be controlled indirectly by the clock, through as-yet uncharacterized factors acting downstream. Alternatively, it may represent the equivalent of an unmasking phenomenon, in which normal cyclic light exposure would lead to suppression of this surge, analogous to melatonin suppression by light. The situation appears quite different in LL, where we observed sizeable phase shifts, but persistent rhythmicity, in clock and photopigment gene expression profiles, as well as scrambled rod and cone phagocytosis with a loss in rhythmicity [20,21]. We propose that the clock somehow becomes uncoupled from the phagocytic control pathway, possibly through inappropriate timing of signaling events. Under such conditions, the capacity of light to directly activate phagocytosis [20], together with increased turnover (at least for cones) triggers phagocytosis across the 24 h period. In short, it is likely that complex dynamic processes such as OS recycling require input from both clock-driven signals and ambient light levels.

In conclusion, examination of the daily transcription profiles of several circadian clock genes, visual pigment, and selected PR genes shows they are under circadian control in a diurnal mammal, tightly synchronized among one another (morning maxima, except *Aanat*). A single 24 h cycle of constant light is sufficient to greatly modify these profiles. The results thus demonstrate strong circadian regulation of both circadian clock genes and several putative outputs, and underscore the dramatic consequences of altered lighting regimes. The latter implies that similar processes may occur in humans during nightshift work, in which nonappropriate (in terms of retinal physiology and circadian regulation) lighting may interfere with normal PR function which could have repercussions for cognitive processes [71]. In this respect, it has been shown recently that nocturnal lighting in a closely related diurnal species, *Arvicanthis niloticus*, leads to appearance of depressive-like behaviors [72].
